# Shift in Candidemia Epidemiology and Emerging Fluconazole Resistance in *Candida parapsilosis*: A Post-Pandemic Cohort Study in a Colombian High-Complexity Teaching Hospital †

**DOI:** 10.3390/jof12070457

**Published:** 2026-06-23

**Authors:** Jenny Patricia Muñoz-Lombo, William David Cardales-Arizal, Raúl Andrés Vallejo-Serna, Indira Berrio

**Affiliations:** 1 Infectious Diseases Department, Hospital Universitario del Valle “Evaristo García”, Cali 760043, Colombia; 2 Infectious Diseases Department, Universidad del Valle, Cali 760043, Colombia; raulvallejomd@gmail.com; 3 Internal Medicine Department, Universidad del Valle, Cali 760043, Colombia; 4 Pathology Department, Universidad del Valle, Cali 760043, Colombia; william.cardales@correounivalle.edu.co; 5 Infectious Diseases Department, Hospital General de Medellín, Medellín 050015, Colombia; indiraberriom@hotmail.com; 6 Basic and Applied Microbiology Research Group (MICROBA), School of Microbiology, Universidad de Antioquia, Medellín 050010, Colombia

**Keywords:** candidemia, *Candida parapsilosis*, fluconazole resistance, antifungal stewardship, invasive fungal infection, Latin America, critical illness, post-pandemic

## Abstract

**Background:** Candidemia remains a significant public health challenge, with increasing resistance. Contemporary post-pandemic data from high-complexity Latin American hospitals are scarce. **Methods**: A retrospective study (2022–2023) was conducted in adults with candidemia at a high-complexity Colombian university hospital. Species identification and susceptibility were analyzed using VITEK^®^ 2 and Clinical and Laboratory Standards Institute (CLSI) criteria. Survival was estimated using Kaplan–Meier analysis. **Results:** Of 3483 blood cultures, 109 episodes were identified. The incidence was 1.13/1000 admissions (5.96/1000 in the Intensive care unit—ICU). Species other than *Candida albicans* predominated (61.5%), mainly *C. tropicalis* (22.9%) and *C. parapsilosis* (22.0%). Alarmingly, 28.6% of *C. parapsilosis* isolates were resistant to fluconazole. Consultation with an infectious diseases service was performed in 72.5% of cases, with a significantly higher rate among survivors (*p* < 0.05). Overall mortality was 52.3%, while 30-day mortality reached 42.2%. ICU patients had a cumulative mortality rate of 50% by day 30. **Conclusions:** Post-pandemic candidemia shows shifting species and high resistance. Key priorities include expert infectious disease consultation to optimize outcomes in non-neutropenic patients and strengthening laboratory capacity for identification and susceptibility testing to monitor rising resistance and guide effective institutional antifungal policies.

## 1. Introduction

*Candida* spp. rank among the highest clinical impact human fungal pathogens, responsible for an estimated 400,000 life-threatening invasive infections annually worldwide [1]. As the fourth most common cause of healthcare-associated bloodstream infections, candidemia exacts a substantial toll in terms of morbidity, attributable mortality (30–60%), and healthcare costs—particularly in tertiary-care settings where the density of immunocompromising exposures is highest [2].

Of the more than 200 described *Candida* species, six account for the vast majority of invasive disease: *C. albicans*, *Nakaseomyces glabrata* (formerly *C. glabrata*), *C. tropicalis*, *C. parapsilosis*, *Pichia kudriavzevii* (formerly *C. krusei*), and the emerging *C. auris* [3,4]. Over the past two decades, a progressive epidemiological transition has occurred globally, with non-albicans species increasingly displacing *C. albicans* as the predominant cause of candidemia, a shift with direct implications for empirical antifungal selection [5].

In Latin America, Colombia historically reports among the highest candidemia incidences in the region (up to 1.9 episodes per 1000 admissions), with ICU-based rates frequently exceeding 40% case fatality [6,7]. Alongside this epidemiological burden, antifungal resistance—particularly fluconazole resistance in *C. parapsilosis*—has emerged as a critical concern in tertiary referral hospitals across the subcontinent [8,9]. The COVID-19 pandemic further disrupted fungal ecology, ICU occupancy, and antimicrobial consumption patterns, with downstream effects on candidemia incidence and species distribution that have not yet been fully characterized [10].

We conducted this study to address this gap and provide practical evidence regarding the incidence, demographic, clinical, and microbiological characteristics of candidemia in a high-complexity university hospital in Cali, Colombia, identifying patterns of antifungal susceptibility as well as the association between specific *Candida* species and adult mortality.

## 2. Materials and Methods

Study Design and Setting: This was a retrospective, single-center cohort study conducted at Hospital Universitario del Valle Evaristo Garcia, a 492-bed public high-complexity teaching hospital in Cali, serving as the primary referral center for southwestern Colombia. The hospital maintains 74 ICU beds and processes approximately 244,000 admissions annually. The study period covered 1 January 2022, through 31 December 2023.

Case Identification and Eligibility: Episodes of candidemia were identified retrospectively from the WHONET microbiology database, which captures all blood culture results processed by the hospital’s clinical microbiology laboratory. All hospitalized adults (≥18 years) with at least one blood culture positive for *Candida* spp. were considered for inclusion. Episodes were excluded if the bloodstream infection was caused by fungi other than *Candida* spp. When a patient experienced multiple episodes, a new episode was defined as isolation of *Candida* spp. more than 30 days after the initial episode, with documented clearance (negative follow-up blood culture). For survival analyses, only the final episode per patient was included.

Microbiological Methods: Blood cultures were incubated for a mean of 96 h, with extended incubation when clinical suspicion was high. Species identification and antifungal susceptibility testing were performed using the VITEK^®^ automated system (bioMérieux, Marcy-l’Étoile, France version 9.02.4). Minimum inhibitory concentrations (MICs) were interpreted according to the Clinical and Laboratory Standards Institute (CLSI) M27 interpretive breakpoints valid up to 2024, aligned with our laboratory’s continuous quality update protocol. *Pichia kudriavzevii* (formerly *Candida krusei*) isolates were classified as intrinsically resistant to fluconazole per CLSI criteria. Additionally, *Candida auris* isolates lacked susceptibility data due to the absence of validated commercial reagents at the time of testing.

Clinical Variables and Outcome Definitions: Variables collected included demographics, comorbidities, ICU vs. non-ICU location at diagnosis, length of hospital stay prior to candidemia, prior antimicrobial and antifungal exposure and specific risk factors or severity indexes (prolonged antibiotic exposure, defined as use within 3 months prior to candidemia diagnosis, length of stay prior to diagnosis, absolute neutrophil count, mechanical ventilation, parenteral nutrition, recent surgery, central venous catheterization, APACHE II score and *Candida* Score). Additionally, variables regarding clinical management and outcomes were recorded, including initial antifungal therapy type and timing, follow-up blood cultures, infectious diseases consultation, ophthalmologic evaluation, and echocardiography. Three mortality endpoints were defined: overall in-hospital mortality (death from any cause during hospitalization), 30-day mortality (death within 30 days of candidemia diagnosis), and candidemia-attributable mortality (death directly adjudicated through clinical chart review in the absence of another identifiable cause).

Statistical Analysis: Continuous variables are summarized as medians with interquartile ranges (IQR) or means ± standard deviations, as appropriate. Categorical variables are expressed as proportions. Distribution normality was assessed using the Kolmogorov–Smirnov test. Comparisons between survivors and non-survivors used the Mann–Whitney U test for continuous variables and chi-square or Fisher’s exact test for categorical variables, with *p* ≤ 0.05 considered statistically significant. Candidemia incidence was calculated as positive-for-*Candida* blood cultures per 1000 hospital admissions, overall and stratified by ICU. Time-to-event analyses for overall, 30-day, and candidemia-attributable mortality were performed using the Kaplan–Meier method; groups were compared using the log-rank test, stratified by *Candida* species and hospital location at diagnosis. All analyses were performed in STATA^®^ software version 17 (StataCorp LLC, College Station, TX, USA).

Ethical Considerations: This study was approved by the Ethics Committee of Hospital Universitario del Valle Evaristo Garcia and classified as minimal-risk research under Colombian Ministry of Health Resolution 8430 of 1993 and the Declaration of Helsinki. Informed consent was waived given the retrospective nature of the study and use of routinely collected, de-identified clinical data.

## 3. Results

### 3.1. Incidence and Study Population

**Figure 1:**
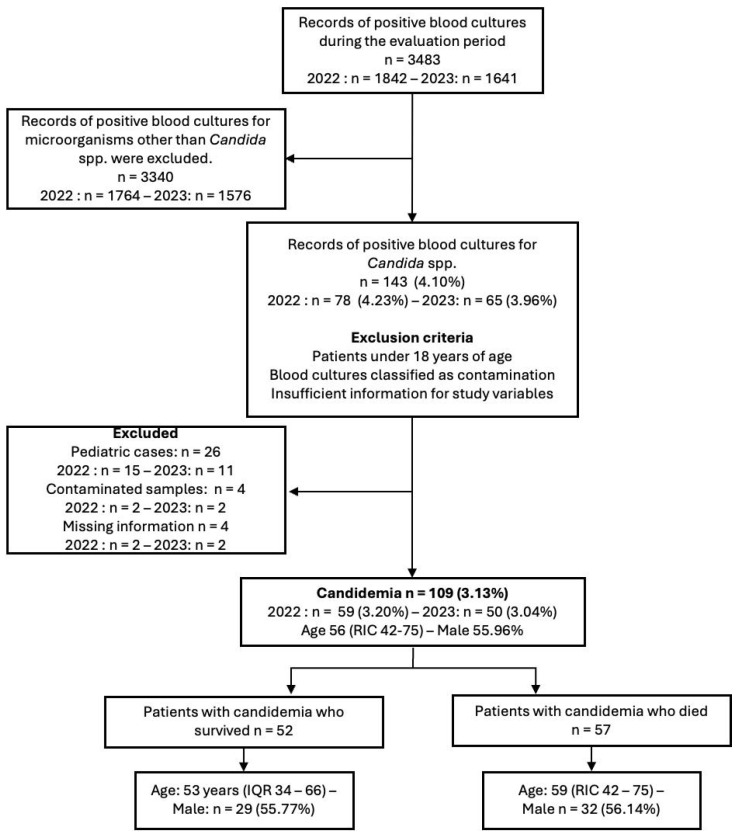
Study flow diagram. Flow diagram illustrating the total number of blood cultures performed, episodes with *Candida* spp. isolated, excluded cases by reason, and the final cohort of 109 candidemia episodes included in the analysis, 2022–2023.

Of 3483 blood cultures performed between January 2022 and December 2023, 143 episodes yielded *Candida* spp.; 109 met eligibility criteria and are included herein (Figure 1). Candidemia accounted for 3.13% of all positive blood cultures (3.20% in 2022; 3.04% in 2023). Overall incidence was 1.13 episodes/1000 admissions (1.35 in 2022; 0.95 in 2023); ICU-specific incidence was 5.96/1000 ICU admissions (7.93 in 2022; 4.50 in 2023).

Baseline characteristics are summarized in Table 1. Median age was 56 years (IQR 42–75); 55.96% of patients were male. At least one comorbidity was present in 60.6% of patients. The most frequent comorbidities were diabetes mellitus (23.9%), chronic kidney disease (22.9%), and renal replacement therapy requirement (28.4%)—the last associated with significantly higher mortality (22 non-survivors vs. 9 survivors, *p* < 0.05). No patient had undergone hematopoietic stem cell transplantation; one had prior solid organ transplantation (renal).

### 3.2. The Critically Ill Host: Candidemia as a Severity Marker Rather than an Isolated Event

A cardinal feature of this cohort was the substantial majority of candidemia in critically ill non-neutropenic patients. At the time of diagnosis, 76.1% of patients were admitted to the ICU, with a mean APACHE II score of 16.7 ± 7.2. The median *Candida* score was 2.0 (IQR 1.9–3.0). Importantly, neither APACHE II nor *Candida* scores differed significantly between survivors and non-survivors. In this population, candidemia emerged across the full spectrum of critical illness severity.

No cases of severe neutropenia were reported. Paradoxically, patients who did not survive had significantly higher neutrophil counts than survivors (11,360 [IQR 7340–17,840] vs. 8590 [IQR 5395–12,635] cells/µL; *p* < 0.05). Similarly, prolonged exposure to broad-spectrum antibiotics (present in 96.3% of patients) and the presence of central venous catheters (84.4%) were the predominant risk factors rather than the classic paradigm of neutropenia associated with hematologic malignancies.

No cases of severe neutropenia were reported. Paradoxically, patients who did not survive had significantly higher neutrophil counts than survivors (11,360 [IQR 7340–17,840] vs. 8590 [IQR 5395–12,635] cells/µL; *p* < 0.05). Similarly, prolonged exposure to broad-spectrum antibiotics (present in 96.3% of patients) and device-related colonization (central venous catheter in 84.4%) were observed, rather than the classic paradigm of neutropenia associated with hematologic malignancies.

Prior surgery was documented in approximately half of the patients, being significantly more frequent among those who did not survive (22 vs. 33; *p* < 0.05), with a median hospital stay prior to candidemia of 19 days (IQR 6.6–33.5), with 70.64% of episodes occurring during the first 30 days of hospitalization, which underlines the role of cumulative healthcare exposure as the main incubator of this infection.

### 3.3. Shifting Fungal Ecology: Predominance of Non-Albicans Species

**Figure 2:**
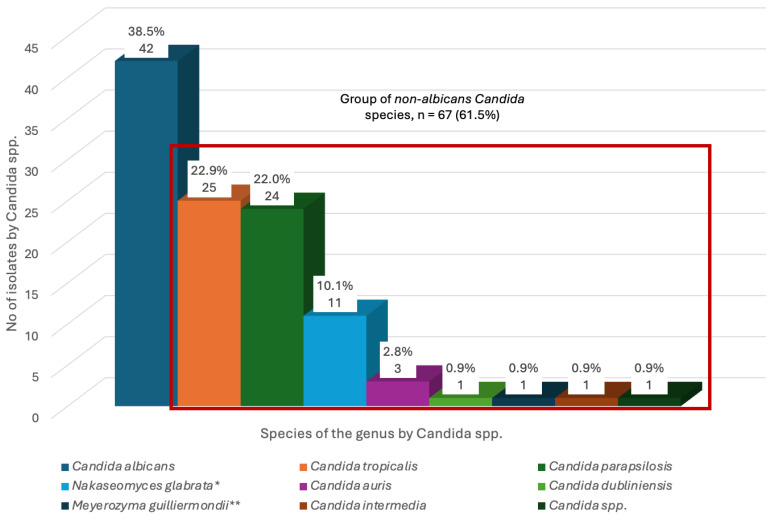
Species distribution. Bar chart depicting the relative frequency of *Candida* spp. isolated from bloodstream infections in adult patients during the study period. Non-*albicans* species collectively accounted for 61.5% of isolates. *Candida glabrata* *; *Candida guilliermondii* **.

Non-*Candida albicans* species accounted for 61.47% of all bloodstream isolates (Figure 2). The most frequently identified pathogen was *C. tropicalis* (22.9%), followed by *C. parapsilosis* (22.0%), *Nakaseomyces glabrata* (formerly *C. glabrata*) (10.1%), and *C. albicans* (38.5%). Rare pathogenic species—*C. dubliniensis*, *C. intermedia*, and *Meyerozyma guilliermondii* (formerly *C. guilliermondii*)—were each identified in a single episode. Three episodes (2.8%) were caused by C. auris (two in 2022, one in 2023); antifungal susceptibility data were unavailable for these isolates due to the absence of validated commercial reagents at our institution.

### 3.4. Antifungal Resistance Dynamics: Alarming Fluconazole Non-Susceptibility in C. parapsilosis

**Figure 3:**
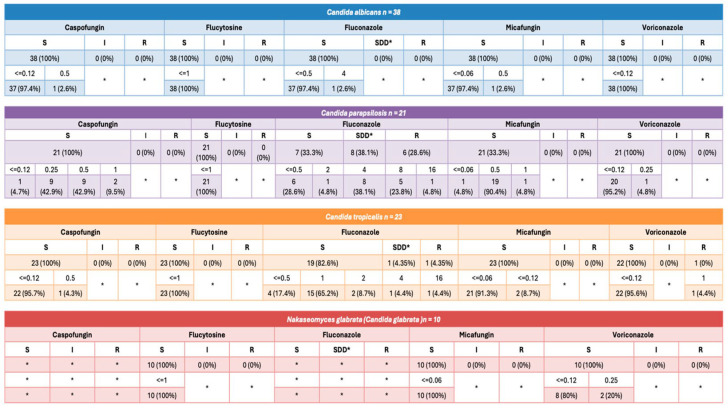
Distribution of the most common *Candida* sensitivity profiles by antifungal and MIC in a Colombian high-complexity teaching hospital. * S: Susceptible; SDD: Susceptible dose-dependent; I: intermediate; R: resistant.

**Figure 4:**
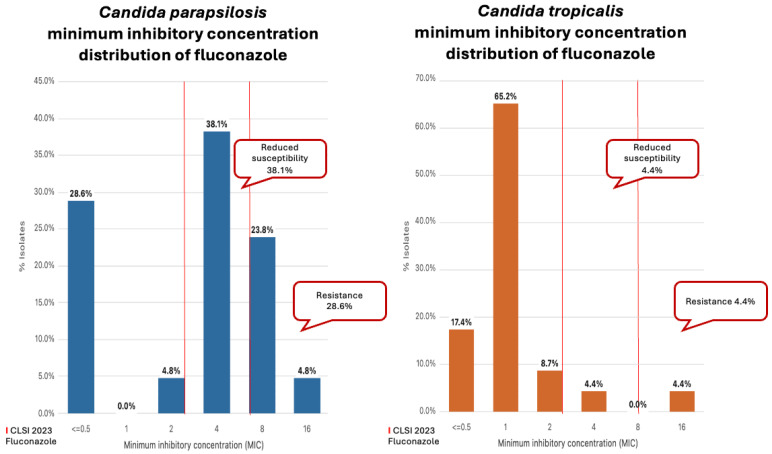
Fluconazole MIC distribution for *C. parapsilosis* and *C. tropicalis.* Distribution of minimum inhibitory concentrations (MICs) for fluconazole among *C. parapsilosis* (*n* = 21) and *C. tropicalis* (*n* = 23) isolates. CLSI breakpoints are indicated by vertical red lines. Proportions classified as susceptible, susceptible dose-dependent/intermediate, and resistant are annotated.

Antifungal susceptibility analysis revealed universal flucytosine susceptibility across all tested species. In contrast, fluconazole susceptibility was markedly compromised among the two most prevalent non-albicans species. *C. parapsilosis* exhibited the most concerning resistance profile: 38.1% of isolates showed reduced fluconazole susceptibility, of which 28.6% were fully resistant (Figure 3 and Figure 4). Among *C. tropicalis*, 4.4% of isolates showed reduced susceptibility and 4.4% were classified as resistant to fluconazole.

### 3.5. Polymicrobial Bloodstream Infection

Concomitant bacteremia was identified in 8.26% of episodes. Among polymicrobial episodes, coagulase-negative *Staphylococci* predominated (44.44%), followed by *Klebsiella pneumoniae* (22.22%) and *Enterococcus faecium*, *Pseudomonas aeruginosa*, and *Stenotrophomonas maltophilia* (11.11% each). No episode involved co-isolation of more than one *Candida* species (Table 2).

### 3.6. Treatment Patterns and the Role of Infectious Diseases Consultation

Antifungal therapy was initiated a median of 1.4 days after microbiological confirmation of candidemia (IQR 0.2–2.4). Specifically, 44% of patients started treatment within the first 24 h, and a cumulative 66% within 48 h. This indicates that nearly two-thirds of the cohort received therapy in less than two days, with minor delays primarily driven by ICU logistical and administrative procedures. Echinocandins were the preferred initial therapy, with caspofungin being the most prescribed agent (65.14% of cases) due to its exclusive availability in our institutional formulary. Antifungal therapy was subsequently modified in 39.4% of patients, predominantly as a de-escalation strategy to fluconazole after species identification and the clearance of fungemia. No antifungal-related adverse events were documented (Table 2).

While our institution was actively implementing a protocol recommending mandatory Infectious Disease (ID) evaluation for all candidemia cases, overall adherence reached 72.5% and was significantly more frequent among survivors (56.1%) (Table 2). The statistically significant higher survival rate observed in the consulted group underscores the impact of a comprehensive clinical evaluation by an ID specialist. This intervention optimizes diagnostic and therapeutic guidance, facilitating the early identification of complications and the timely implementation of advanced clinical interventions.

Ophthalmologic evaluation was performed in 45% of patients (two cases of *Candida* endophthalmitis identified), and echocardiography in 33% (three cases of *Candida* endocarditis, none fatal) (Table 2). Although both studies are routinely recommended by the ID consultation team to screen for metastatic infection, full completion was limited by logistical barriers, including patient mortality, restricted specialist availability at the bedside, and instances of non-adherence by the primary medical services to ID recommendations.

Follow-up blood cultures were performed in 74 patients, with candidemia clearance documented in 87.8% at the first control (median 5 days, IQR 3–7). The remaining patients did not undergo follow-up cultures primarily due to early mortality or, to a lesser extent, non-adherence to the institutional protocol by the primary medical services (Table 2).

### 3.7. Mortality Analysis: Disentangling Global from Candidemia-Attributable Death

**Figure 5:**
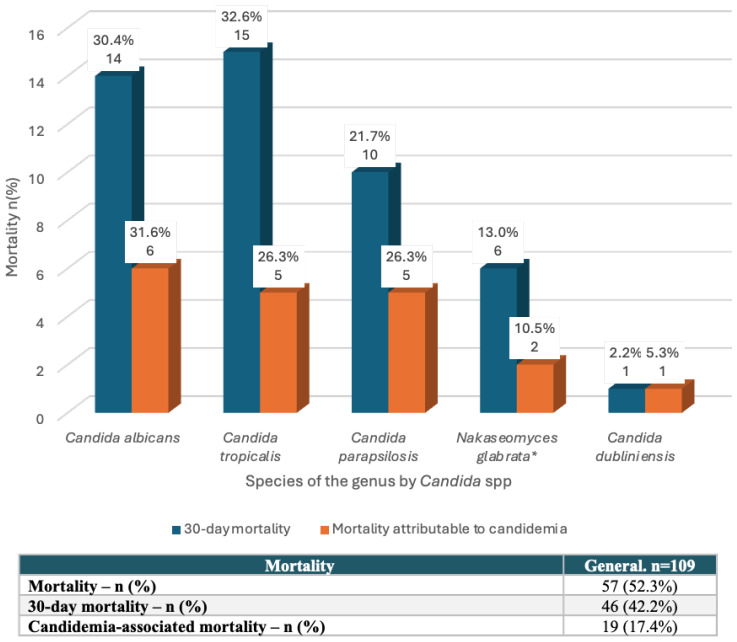
Summary of mortality outcomes. Bar chart depicting overall in-hospital mortality (52.29%), 30-day mortality (42.20%), and candidemia-attributable mortality (17.43%) for the full cohort, with species-stratified breakdown. * *Candida glabrata*.

**Figure 6:**
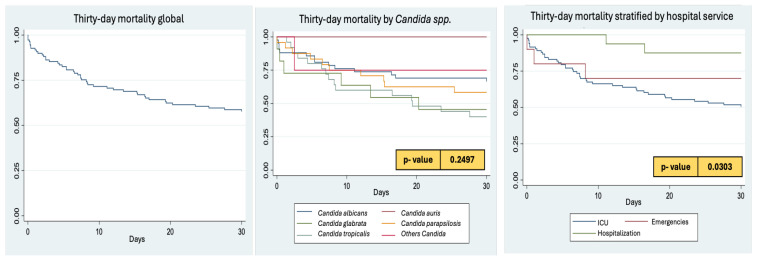
Thirty-day mortality: Kaplan–Meier survival curves for 30-day mortality stratified by hospital service at the time of candidemia diagnosis.

Overall in-hospital mortality was 52.3% (57/109), 30-day mortality was 42.2% (46/109), and candidemia-attributable mortality was 17.4% (19/109). (Figure 5). Kaplan–Meier survival analysis demonstrated that approximately 30% of patients died within the first 10 days of candidemia diagnosis (Figure 6). By day 30, ICU patients reached 50% cumulative mortality (log-rank *p* < 0.05 vs. non-ICU patients), while no statistically significant differences in 30-day mortality were observed according to *Candida* species.

## 4. Discussion

To our knowledge, this study represents the first contemporary post-pandemic characterization of candidemia at a high-complexity teaching hospital in southwestern Colombia, and one of the most recent institutional cohorts from Latin America to integrate species distribution, antifungal resistance profiling, clinical severity analysis, and mortality attribution within a unified analytical framework.

In Colombia, invasive fungal infections represent a significant burden, historically, *Candida* spp. have been the most frequently isolated yeast in bloodstream infections (BSIs) [6,11] While our observed incidence was slightly lower than that of historical national cohorts [7,12], it is consistent with recent data from Latin America [13,14]. This could reflect the evolution of post-pandemic healthcare dynamics and changes in the composition of patient cases, rather than a true reduction in the disease burden. It is worth noting that the predominance of non-neutropenic patients with chronic comorbidities—particularly diabetes and kidney disease—which is consistent with previous reports of candidemia in different contexts [15,16,17], reinforces the idea that candidemia in our context is due to cumulative exposure to medical care, metabolic dysfunction, microbiome dysbiosis, and device-related colonization [18].

The clinical severity of our cohort is underscored by elevated APACHE II scores—associated with critical illness and increased mortality risk in ICU populations [19], and comparable to those reported in previous local studies [20]—alongside a high proportion of ICU admissions at the time of diagnosis. This positions candidemia more as an indicator of cumulative physiological vulnerability rather than a discrete, predictable complication or an isolated opportunistic event. Interestingly, the median *Candida* score remained below traditional risk thresholds [21], highlighting the limited discriminatory performance of existing risk scores in contemporary ICU populations.

No cases of neutropenia were identified in this cohort; instead, higher neutrophil counts were observed among non-survivors. This departs from the classic paradigm associating candidemia with neutropenia or profound immunosuppression [22]. In our predominantly non-neutropenic, critically ill population, neutrophilia likely reflects an exaggerated systemic inflammatory response rather than effective host defense. As demonstrated in sepsis studies, elevated neutrophil counts can coexist with impaired function and immune dysregulation [23,24]. Consequently, the neutrophilia seen in non-survivors serves as a marker of disease severity and inflammatory burden, suggesting distinct pathophysiological mechanisms in the non-hematological ICU milieu [25].

Bloodstream infections may be polymicrobial, occasionally involving synchronous bacteremia or even multiple *Candida* species within the same culture. Although it has been reported that up to 23% of candidemia episodes—approximately one in four—present with concurrent bacteremia [26], our cohort demonstrated a markedly lower frequency of only 8.26%. Furthermore, while literature suggests that up to 3% of cases may involve multiple *Candida* species, no such mixed fungal infections were observed in our study.

Regarding bacterial co-pathogens, the literature presents discordant data; some reports suggest a lower association with *Staphylococcus aureus* and coagulase-negative staphylococci (CoNS) in polymicrobial candidemia [26]. Conversely, other studies identify CoNS as the predominant co-pathogen, accounting for 23% of cases [27]. Our findings align with the latter, as CoNS represented 44.44% of the bacterial microorganisms identified in our series. The low rate of concomitant pathogens in our cohort reduces potential confounding from bacterial sepsis, strengthening the clinical attribution of outcomes to candidemia itself.

A pivotal finding in our cohort is the clear predominance of non-*albicans* species. While *Candida albicans* has historically led regional reports [20,28,29], our data confirm an accelerating trend toward *C. tropicalis* and *C. parapsilosis.* This epidemiological shift carries profound therapeutic implications, particularly given the alarming level of fluconazole resistance documented in *C. parapsilosis* (28.6% fully resistant; 38.1% with reduced susceptibility). These figures render empirical azole therapy untenable for this species at our center and mirror recent Colombian studies in Bogotá (38%) and Medellín (30%) that describe a rising resistance crisis [8,9].

Antimicrobial resistance in *C. parapsilosis* represents a critical public health concern [30]. Historically, fluconazole resistance in *C. parapsilosis* was an exceptional finding. Before 2010, resistant strains were identified only occasionally; however, in recent years, resistance rates have surged beyond 10%, even outpacing susceptible strains in certain clinical settings [31]. This phenomenon is multifaceted, involving molecular mechanisms such as drug-target modification [32]. Although clinical factors like diabetes mellitus have been previously associated with resistant strains [33], only 13.6% of our patients presented this comorbidity. This discrepancy suggests that local antifungal selective pressure or nosocomial clonal transmission may play a more significant role. Consequently, these data mandate an “echinocandin-first” approach and underscore the urgent need for molecular epidemiological studies to investigate potential hospital-acquired outbreaks.

Regarding the timing of therapy initiation, our cohort demonstrated a shorter median time compared to previous reports, reflecting an institutional focus on early detection and prompt treatment. Similar optimization efforts have been documented in Brazil, where the time to treatment initiation was reduced from 4 to 2 days [13]. In our study, empirical therapy was initiated within the first 24 h in 44% of patients, reaching 66% by 48 h. These figures are highly comparable to results from other high-complexity centers in the region [34].

Overall mortality was 52.29%, with 42.2% occurring within 30 days and nearly one-third of deaths within the first 10 days post-diagnosis. While mortality did not differ significantly by *Candida* species, it was markedly higher in ICU patients, reaching 50% at 30 days. These findings align with Colombian and Latin American cohorts, where baseline severity of illness—rather than species distribution—is the primary driver of mortality [13,14,35]. Thus, in this setting, candidemia serves as a marker of advanced critical illness.

The almost 35-percentage-point discrepancy between overall mortality (52.3%) and attributable mortality (17.4%) underscores a crucial distinction: while candidemia remains a devastating complication, the majority of deaths likely reflect underlying clinical frailty.

Consistent with global evidence, our data demonstrated a statistically significant higher survival rate within the group that received an infectious disease consultation (IDC), underscoring the profound clinical impact of a comprehensive specialist evaluation. In our clinical setting, IDC directly optimized diagnostic and therapeutic guidance by ensuring appropriate, timely antifungal selection, facilitating early source control (such as central venous catheter removal), and structuring the systematic search for deep-seated complications via ophthalmologic and echocardiographic evaluations. This marked survival benefit aligns with previous cohorts where 90-day mortality was substantially lower in patients receiving specialized consultation (29% vs. 51%; *p* < 0.0001) [36]. Collectively, these findings provide a compelling argument for specialized intervention and reinforce the premise that mandatory IDC should be established as a key quality-of-care indicator in the management of candidemia.

Although ocular involvement remains a relatively rare complication, its diagnosis is pivotal for tailoring therapeutic strategies. In a recent cohort, 76.4% of patients underwent fundoscopy—a rate significantly higher than the 45% observed in our study—yielding an ocular candidiasis prevalence of 4.9% (15/307) [37]. This aligns closely with our 4.1% (2/29) incidence. Despite occurring in fewer than 5% of cases, fundoscopy remains a cornerstone of international management guidelines [38] due to its profound impact on clinical decision-making.

The findings of this study underscore the critical importance of implementing robust Antifungal Stewardship (AFS) programs in high-complexity settings, particularly within Intensive Care Units. Prior to this investigation, institutional antimicrobial stewardship initiatives focused predominantly on antibacterial agents, leaving antifungal consumption metrics, such as Defined Daily Doses (DDDs), largely unmonitored. Paradoxically, the high reliance on empiric therapies and the shifting epidemiological landscape revealed here served as the fundamental driver for integrating AFS into our hospital’s clinical agenda. Establishing routine audit-and-feedback mechanisms for antifungal prescriptions is essential not only to optimize clinical response times but also to curb the selection pressure driving emerging azole resistance in *Candida parapsilosis*.

### Strengths and Limitations

This study benefits from a detailed clinical–microbiological characterization and a low rate of polymicrobial bloodstream infections, which strengthens the attribution of clinical outcomes. However, several limitations merit acknowledgment. The primary scope was oriented toward defining the baseline incidence and landscapes of candidemia in our institution; consequently, mortality was evaluated across *Candida* species rather than through a direct correlation with antifungal susceptibility profiles *per se*. While this leaves a clear avenue for future prospective research using competing risks analysis, our data yielded a critical epidemiological finding: *Candida parapsilosis* emerged as the third leading pathogen associated with both 30-day and candidemia-attributable mortality, highlighting its substantial clinical impact. Finally, the retrospective, single-center design limits generalizability, and the lack of molecular typing for *C. parapsilosis* prevents us from distinguishing between clonal spread and convergent resistance acquisition.

## 5. Conclusions

Candidemia in this post-pandemic era has evolved into a complication of the modern ICU milieu, characterized by a predominance of non-*Candida albicans* species, high fluconazole resistance in *C. parapsilosis*, near-exclusive occurrence in critically ill non-neutropenic patients, and a large gap between overall and attributable mortality.

Our data emphasizes that managing this infection requires more than just antifungal administration. These findings collectively support three institutional priorities:

(1) Empirical echinocandin therapy for all ICU-acquired candidemia pending susceptibility results.
(2) Molecular epidemiological investigation of *C. parapsilosis* to characterize the local resistance landscape and guide infection control measures.
(3) Mandatory ID consultation as a formalized component of antifungal stewardship for all candidemia episodes.

Future prospective multicenter studies incorporating competing risks methodology and molecular typing are needed to validate these findings and inform regional antifungal policy.

## Figures and Tables

**Table 1:** Epidemiological characterization of adult patients with candidemia between 2022–2023 in a Colombian high-complexity teaching hospital.

Characteristics	Overall, *n* = 109	Mortality ^a^		*p*-Value
		No, *n* = 52	Yes, *n* = 57	
Age *	56 (42–75)	53 (34–66)	59 (42–75)	0.065
Male sex—no. (%)	61 (56)	29 (55.8)	32 (56.1)	0.969
Total length of stay	45 (22–82)	69 (39.5–111)	29 (17–53)	0
Service at time of diagnosis—no. (%)				
ICU	83 (76.1)	34 (65.4)	49 (86)	0.074
General ward	13 (11.9)	9 (17.3)	4 (7)	
Emergency Department	10 (9.2)	7 (13.5)	3 (5.3)	
Hemato-Oncology	2 (1.8)	1 (1.9)	1 (1.8)	
Burn unit	1 (0.9)	1 (1.9)	0 (0)	
Comorbidities—no. (%)	66 (60.6)	31 (59.6)	35 (61.4)	0.849
Diabetes mellitus	26 (23.9)	9 (17.3)	17 (29.8)	0.126
Solid tumor	19 (17.4)	12 (23.1)	7 (12.3)	0.138
Hematologic malignancy	6 (5.5)	3 (5.8)	3 (5.3)	1
Hematopoietic stem cell transplant (HSCT)	0 (0)	0 (0)	0 (0)	NA
Solid organ transplant (SOT)	1 (0.9)	1 (1.9)	0 (0)	0.477
Chronic kidney disease (CKD)	25 (22.9)	11 (21.2)	14 (24.6)	0.673
Hemodialysis	31 (28.4)	9 (17.3)	22 (38.6)	0.014
Liver disease	7 (6.4)	3 (5.8)	4 (7)	0.791
HIV	4 (3.7)	3 (5.8)	1 (1.8)	0.346
Burn injury/Major burns	4 (3.7)	3 (5.8)	1 (1.8)	0.346
Intravenous drug user	6 (5.5)	4 (7.7)	2 (3.5)	0.422
Steroid-induced immunosuppression	3 (2.8)	2 (3.8)	1 (1.8)	0.605
LOS at time of diagnosis	19 (7–34)	20 (6–42)	15 (7–29)	0.238
Candidemia—no. (%)				
2022	59 (54.1)	22 (42.3)	37 (64.9)	0.018
2023	50 (45.9)	30 (57.7)	20 (35.1)	
Risk factors for the development of candidemia				
APACHE II *	16.7 ± 7.2	15.2 ± 7.4	17.7 ± 6.9	0.1094
*Candida* score **	2 (1.9–3)	2 (0–3)	2(2–3)	0.1014
Prolonged exposure to antibiotics	105 (96.3)	49 (96.6)	50(96.2)	1
Central venous catheter—no. (%)	92 (84.4	92 (84.4)	43 (82.7)	0.638
Mechanical ventilation—no. (%)	65 (59.6)	65 (59.6)	20 (38.5)	0
Parenteral nutrition (PN)—no. (%)	16 (14.7)	16 (14.7)	9 (17.3)	0.459
Surgery—no. (%)	55 (50.5)	55 (50.5)	33 (63.5)	0.01
Neutrophil count—no. (%)	9730 (6030–16,080)	8590 (5395–12,635)	11,360 (7340–17,840)	0.0158

Source = Author’s data. APACHE II: Acute Physiology and Chronic Health Assessment II; ICU: Intensive Care Unit; HIV: Human immunodeficiency virus; N.A.: not applicable: LOS: Length of stay to candidemia onset. ^a^: In-hospital mortality. * Median with IQR = Interquartile range. ** Mean with SD = Standard deviation.

**Table 2:** Microbiological and antifungal characterization of adult patients with candidemia between 2022–2023 in a Colombian high-complexity teaching hospital.

Characteristics	Overall, *n* = 109	Mortality		*p*-Value
		No, *n* = 52	Yes, *n* = 57	
Microorganism—no. (%)				
*Candida albicans*	42 (38.5)	23 (44.2)	19 (33.3)	0.556
*Candidozyma auris* (*Candida auris*)	3 (2.8)	1 (1.9)	2 (3.5)	
*Candida dubliniensis*	1 (0.9)	0 (0)	1 (1.8)	
*Nakaseomyces glabrata* (*Candida glabrata*)	11 (10.1)	5 (9.6)	6 (10.5)	
*Meyerozyma guilliermondii* (*Candida guilliermondii*).	1 (0.9)	1 (1.9)	0 (0.0)	
*Candida intermedia*	1 (0.9)	1 (1.9)	0 (0.0)	
*Candida parapsilosis*	24 (22.0)	12 (23.1)	12 (23.1)	
*Candida* spp.	1 (0.9)	1 (1.9)	0 (0.0)	
*Candida tropicalis*	25 (22.9)	8 (15.4)	17 (29.8)	
Identification of non-*Candida* spp. microorganism—no. (%)	9 (8.3)	9 (8.3)	3 (5.8)	0.493
*Enterococcus faecium*	1 (0.9)	1 (1.9)	0 (0)	1
*Klebsiella pneumoniae*	2 (1.8)	1 (1.9)	1 (1.8)	
*Pseudomonas aeruginosa*	1 (0.9)	0 (0)	1 (1.8)	
Coagulase-negative *Staphylococcus* (CoNS)	1 (0.9)	0 (0)	1 (1.8)	
*Staphylococcus epidermidis*	1 (0.9)	0 (0)	1 (1.8)	
*Staphylococcus haemolyticus*	1 (0.9)	0 (0)	1 (1.8)	
*Staphylococcus hominis*	1 (0.9)	1 (1.9)	0 (0)	
*Stenotrophomonas maltophilia*	1 (0.9)	0 (0)	1 (1.8)	
Time to initiation of antifungal therapy (days) *	1.4 (0.2–2.4)	1.4 (0.4–2.4)	1.4 (0.1–2.2)	0.327
Initial antifungal therapy—no. (%)				
Caspofungin	71 (65.1)	33 (63.5)	38 (66.7)	0.414
Anidulafungin	1 (0.9)	0 (0)	1 (1.8)	
Fluconazole	25 (22.9)	15 (28.8)	10 (17.5)	
Liposomal Amphotericin B	2 (1.8)	1 (1.9)	1 (1.8)	
Change in antifungal therapy—no. (%)	*n* = 99	*n* = 49	*n* = 50	
No	60 (60,6)	23 (46.9)	37 (74.0)	0.006
Yes	39 (39.4)	26 (53.1)	13 (26.0)	
Reason for therapy change—no. (%)	*n* = 39	*n* = 26	*n* = 13	
De-escalation	27 (69.2)	20 (76.9)	7 (53.8)	0.163
Escalation	12 (30.8)	6 (23.1)	6 (46.2)	
Infectious Diseases consultation—no. (%)	79 (72.5)	47 (90.4)	32 (56.1)	**0.000**
Ophthalmology consultation—no. (%)	49 (45)	36 (69.2)	13 (22.8)	**0.000**
Endophthalmitis—no. (%)	2 (4.1)	2 (5.)	0 (0)	**0.000**
Echocardiogram performed—no. (%)	36 (33)	20 (38.5)	16 (28.1)	1
Endocarditis—no. (%)	3 (8.3)	3 (15.0)	0 (0)	0.316
Clearance of fungemia on first follow-up blood culture—*n* = 74—no. (%)	65 (87.8)	41 (58.1)	24 (29.7)	0.283
Days to follow-up blood culture request *	5 (3–7)	5 (3–6.5)	5 (3–8)	0.973
Hospital length of stay from diagnosis to discharge *	19 (7–50)	40 (19–78)	8 (3–24)	**0.000**

Source = Author’s data. * Median with IQR = Interquartile range.

## Data Availability

The data presented in this study are available upon request from the corresponding author. The data are not publicly available due to institutional privacy and confidentiality policies regarding medical records.

## Abbreviations

The following abbreviations are used in this manuscript:

APACHE II	Acute Physiology and Chronic Health Assessment II
CLSI	Clinical and Laboratory Standards Institute
CoNS	Coagulase-negative *Staphylococci*
COVID-19	Coronavirus Disease 2019
HIV	Human immunodeficiency virus
ICU	Intensive care unit
ID	Infectious diseases
LOS	Length of stay
MICs	Minimum inhibitory concentrations
N.A.	Not applicable
